# Ultra-Processed Food Consumption and Mental Health: A Systematic Review and Meta-Analysis of Observational Studies

**DOI:** 10.3390/nu14132568

**Published:** 2022-06-21

**Authors:** Melissa M. Lane, Elizabeth Gamage, Nikolaj Travica, Thusharika Dissanayaka, Deborah N. Ashtree, Sarah Gauci, Mojtaba Lotfaliany, Adrienne O’Neil, Felice N. Jacka, Wolfgang Marx

**Affiliations:** 1 Food & Mood Centre, The Institute for Mental and Physical Health and Clinical Translation (IMPACT), School of Medicine, Deakin University, Geelong, VIC 3220, Australia; egamage@deakin.edu.au (E.G.); nikolaj.travica@deakin.edu.au (N.T.); t.dissanayaka@deakin.edu.au (T.D.); debbie.ashtree@deakin.edu.au (D.N.A.); sarah.gauci@deakin.edu.au (S.G.); m.lotfalianyabrandabadi@deakin.edu.au (M.L.); adrienne.oneil@deakin.edu.au (A.O.); f.jacka@deakin.edu.au (F.N.J.); wolf.marx@deakin.edu.au (W.M.); 2 Centre for Adolescent Health, Murdoch Children’s Research Institute, Parkville, VIC 3052, Australia; 3 College of Public Health, Medical & Veterinary Sciences, James Cook University, Townsville, QLD 4811, Australia

**Keywords:** major depressive disorder, anxiety, mental disorders, ultra-processed food, NOVA, meta-analysis, nutritional psychiatry, psychiatry

## Abstract

Since previous meta-analyses, which were limited only to depression and by a small number of studies available for inclusion at the time of publication, several additional studies have been published assessing the link between ultra-processed food consumption and depression as well as other mental disorders. We aimed to build on previously conducted reviews to synthesise and meta-analyse the contemporary evidence base and clarify the associations between the consumption of ultra-processed food and mental disorders. A total of 17 observational studies were included (*n* = 385,541); 15 cross-sectional and 2 prospective. Greater ultra-processed food consumption was cross-sectionally associated with increased odds of depressive and anxiety symptoms, both when these outcomes were assessed together (common mental disorder symptoms odds ratio: 1.53, 95%CI 1.43 to 1.63) as well as separately (depressive symptoms odds ratio: 1.44, 95%CI 1.14 to 1.82; and, anxiety symptoms odds ratio: 1.48, 95%CI 1.37 to 1.59). Furthermore, a meta-analysis of prospective studies demonstrated that greater ultra-processed food intake was associated with increased risk of subsequent depression (hazard ratio: 1.22, 95%CI 1.16 to 1.28). While we found evidence for associations between ultra-processed food consumption and adverse mental health, further rigorously designed prospective and experimental studies are needed to better understand causal pathways.

## 1. Introduction

Mental disorders are among the leading causes of global burden, and a recent report by the Global Burden of Disease Study noted that, despite greater availability of treatments (e.g., increase in prescriptions), there has been no reduction in the burden of mental disorders since 1990 [1]. The *Lancet* Commission on global mental health and sustainable development highlighted mental health as a fundamental human right, and called for the improvement and unification of mental health services into the global response to other health priorities [2]. Similarly, there have been recent calls for integrating mental disorders into the “big four” non-communicable disease framework [3,4], alongside cancer, diabetes, respiratory and cardiovascular conditions. Efficacious preventative and treatment strategies by government and global health communities to reduce the burden of mental disorders will require a comprehensive understanding of the factors that may be driving their incidence and prevalence [1].

Poor dietary quality is well established as a potentially modifiable risk factor for mental disorders [5,6]. Historically, the associations of poor dietary quality with mental disorders have largely focused on depression [7,8]. These associations have mostly been examined via diet quality indices or dietary pattern analyses of food items, such as the consumption of red and processed meat and refined grains, or macronutrient content, such as saturated fat and sugar intake [7,8,9,10]. However, emerging evidence implicates different degrees of food processing as a discrete indicator of dietary quality in mental disorders.

The NOVA food classification system was recently developed to enable the categorisation of food items based on distinctive levels of processing [11]. This system includes four incrementally processed groups: (1) unprocessed or minimally processed food, (2) processed culinary ingredients, (3) processed food, and (4) ultra-processed food [11]. NOVA distinguishes ultra-processed foods as industrial formulations generated through compounds extracted, derived or synthesised from food or food substrates [11]. Ultra-processed food items are characterised as containing five or more ingredients, which typically include artificial food additives rarely or never used in home kitchens (e.g., preservatives, colours, texturising agents, and olfactory and taste enhancers). These food items are frequently low priced, convenient, shelf-stable, easily consumed and highly palatable [12]. Recent global estimates demonstrate substantial growth in the types and quantities of ultra-processed foods procured [13,14] and consumed worldwide [15]. Indeed, at the beginning of 2021, we published a systematic review and meta-analysis that showed ultra-processed foods accounted for 17% to 56% (mean of 37%) of total daily energy intake across 28 countries [16]. 

We also reported positive associations between ultra-processed food consumption and 15 chronic non-communicable diseases and related morbidity and mortality, including a meta-analysis on mental disorders [16]. However, the meta-analysis assessing associations of ultra-processed food consumption with mental disorders was limited only to depression and by the number of studies available for inclusion at the time of publication (*n* = 2). Several additional studies have since been published assessing the link between ultra-processed food consumption and depression as well as other mental disorders [17,18,19,20,21,22,23,24,25,26,27,28,29,30,31]. The need for an updated review is further exacerbated by the fact that ultra-processed foods have become increasingly ubiquitous for many food systems globally [13], thus understanding any relationship with mental disorders is critical for understanding the health implications of this trend. Therefore, we aimed to synthesise and provide quantitative analyses of the most up-to-date evidence assessing associations between consumption of ultra-processed food, as defined by the NOVA food classification system, and mental disorders. 

## 2. Methods

### 2.1. Search Strategy

This review was prospectively registered with the International Prospective Register of Systematic Reviews, better known as PROSPERO (ID: CRD42022311620), and has been reported in accordance with the Preferred Reporting Items for Systematic Reviews and Meta-Analyses (PRISMA) guidelines (see Figure S1 for PRISMA flowchart) [32]. Given the NOVA food classification system was developed in 2009 [33], databases (MEDLINE complete, EMBASE and Scopus) were searched from January 2009 to March 2022. Search terms were a combination of free-text terms and controlled vocabulary related to ultra-processed food, NOVA and mental disorders (see Table S1 for search terms across databases). After full-text screening, we searched citations from included studies to find additional relevant studies.

### 2.2. Study Selection, Inclusion and Exclusion Criteria

To be eligible for inclusion, studies met the following criteria: written in English, conducted in humans of any age (clinical and general populations); observational by design; investigated the association between ultra-processed food intake and mental disorders; and compared different levels of ultra-processed food consumption (e.g., lower versus higher) or ultra-processed food versus unprocessed or minimally processed food. Studies were excluded if the NOVA food classification system was not used or if the direct consumption of ultra-processed food was not investigated (e.g., household availability, access to, price of and purchase of ultra-processed food).

To provide the most comprehensive overview of the relationship between ultra-processed food consumption and mental disorders, we included studies that modelled ultra-processed food consumption as either the exposure or outcome. In addition, we included studies where mental disorder parameters were derived from either clinician-rated or self-reported assessments and definitions varied (e.g., presence of common mental disorders, depressive symptoms, depressive mood, diagnosis of depression, anxiety symptoms, anxiety-induced sleep disturbance, etc.). We aimed to synthesise these definitions and maximise the generalisability of our findings by grouping together mental disorder categories based on the Diagnostic and Statistical Manual of Mental Disorders—5th Edition [34], and listing whether mental disorder diagnoses or symptoms that align with mental disorders were investigated. That is, mental disorder categories make up the major themes of our review and results are presented as such. These themes include the common mental disorders (e.g., depression and anxiety, which we assessed together given their common comorbidity [35] as well as separately), trauma and stress-related disorders (e.g., post-traumatic stress disorder and perceived stress), addiction-related disorders (e.g., food addiction and alcohol use disorder) and eating disorders (e.g., anorexia nervosa, bulimia nervosa and binge eating disorder).

### 2.3. Data Extraction

The titles and abstracts of individual studies were screened for eligibility by two authors (EG, NT). After title and abstract screening, two authors (EG, NT) assessed full-text articles for inclusion into the systematic review. At both screening stages, disagreements were resolved by consensus or by a third author (MML) when consensus could not be reached. The following information was extracted in duplicate (EG, NT, TD): author, date, study design, sample size, sample characteristics (including age, % male and exclusion criteria), dietary data characteristics (including how data were collected and analysed, tool(s) used, diet data collection duration, details of NOVA classification), confounding variables and details of mental disorder parameters (how data were collected and analysed, tool(s) used and results). We considered the results presented in the original studies’ abstracts to be the main statistical model, unless otherwise indicated by the authors. The corresponding author(s) of the original papers were contacted in an attempt to retrieve missing information.

### 2.4. Critical Appraisal Assessment

A set of standardised critical appraisal instruments from the Joanna Briggs Institute was used by two authors (DA, SG) to examine the methodological validity of studies included in the review [36]. This set included the Critical Appraisal Checklist for Cross-Sectional and Cohort Studies [36]. Instrument items ask assessors to examine whether the original studies provided adequate information pertaining to population characteristics, exposures, confounders, outcomes, follow-up details (where applicable) and statistical analysis. These instruments are qualitative and allow for an overall appraisal based on the following answers to instrument items: yes, no, unclear or not applicable [36]. We used these appraisals to inform our synthesis and interpretation of the results of studies that met the inclusion criteria described in the prospectively registered protocol.

### 2.5. Data Analysis

Results were pooled in a meta-analysis when studies reported consistent data analysis methods, study designs and mental disorder outcomes. As suggested by Borenstein et al. (2009), when studies reported results in different ways (e.g., prevalence ratios versus odds ratios), summary data (i.e., the number of ‘cases’ versus ‘controls’ in un/exposed groups) were used to compute and then combine the same index for all studies [37]. Hazard ratios and odds ratios with 95% confidence intervals (95% CIs) for binary outcomes were used. Studies not eligible for inclusion in a meta-analysis formed part of the narrative syntheses, which is presented by outcome, population and context characteristics. 

Meta-analyses were conducted using a random-effects model in Comprehensive Meta-Analysis 3.0 software [38]. The I-squared statistic (I^2^) was used to assess between-study heterogeneity [39]. Substantial heterogeneity was defined as an I^2^ value greater than 50% [40]. A one-study-removed sensitivity analysis was conducted to determine whether overall estimates were influenced by outlier studies. 

In line with previous recommendations [41,42,43,44], we examined the sizes of effect estimates and 95% CIs, as well as precise *p*-values, to consider the strength of our findings. Three studies included in the narrative syntheses did not report confidence intervals [17,23,29]. For two of these [23,29], we estimated the confidence intervals by using the estimated effects and *p*-values as per the methods proposed by Altman and Bland (2011) for beta-coefficients [45] and Bishara and Hittner (2017) for Pearson correlation coefficients [46]. These are denoted in the Results section as ‘estimated 95% CIs’. Although the third study reported a beta-coefficient, it was not possible to estimate the confidence interval given an exact *p*-value or measure of variance were not reported [17]. Similarly, four studies [20,25,26,27] did not report *p*-values. We estimated these by using the confidence intervals as per the methods proposed by Altman and Bland (2011) [47]. These are reported in the Results section as ‘estimated *p*…’.

## 3. Results

### 3.1. Search Results

The search strategy yielded 1083 de-duplicated studies that were screened to identify 17 eligible studies for inclusion (see Figure S1 for PRISMA flowchart).

### 3.2. Study Characteristics

A total of 385,541 participants were included in the 17 eligible studies. Sample sizes ranged from 33 to 100,684 participants. Two studies conducted analyses on the same sample of adolescents; one had a sample size of *n* = 100,648 and used an exposure that combined high ultra-processed food intake with high sedentary behaviour [26]; and the other had a sample size of *n* = 99,971 and separated these lifestyle behaviours [25]. Included studies examined associations between ultra-processed food consumption and the common mental disorders (i.e., depression and anxiety, which were assessed together (*N* = 2) and separately (*N* = 8 for depression and *N* = 6 for anxiety)). In addition, studies assessed links between ultra-processed food consumption and other mental disorder parameters, such as trauma and stress (i.e., post-traumatic stress disorder (*N* = 1) and perceived stress (*N* = 3)), addiction (i.e., food addiction (*N* = 2) and alcohol use disorder (*N* = 1)) and eating disorders (i.e., anorexia nervosa, bulimia nervosa and binge eating disorder (*N* = 1)).

A total of 15 cross-sectional and 2 prospectively designed studies (average follow-up of 7.85 years) were included. Eligible studies were conducted in Brazil (*N* = 9), the United States of America (*N* = 2), Italy (*N* = 2), the United Kingdom (*N* = 1), Spain (*N* = 1), France (*N* = 1) and Belgium (*N* = 1). Eligible studies included different age groups, including adults (*N* = 11), adolescents (*N* = 4), children (*N* = 1), and mixed-age groups (adolescents and adults (*N* = 1)). The mean age of all participants was 35.2 years (not including four studies that reported age categories [18,19,20,21]), with a mean age of 44.9 years for adults, 14.5 years for adolescents and 9.6 years for children. In the 16 studies that reported sex proportions, males accounted for 44.5% of the samples combined. Dietary data were either self-reported (*N* = 10) or assessed via interview (*N* = 7), and collected using food-frequency questionnaires (*N* = 10), 24-h dietary recalls (*N* = 6) and diet history over the preceding two weeks (*N* = 1) (see Table 1 below for a detailed summary of study characteristics). 

### 3.3. Details of Exposure Variables and Average Ultra-Processed Food Consumption

Eleven studies modelled ultra-processed food consumption as the exposure variable and mental disorder parameters as the outcome [17,18,19,21,23,24,25,26,30,48,50], with the remaining six modelling associations in the opposite direction [20,22,27,28,29,31]. 

Of the 11 studies that treated ultra-processed food consumption as the exposure variable, 8 coded ultra-processed food intake categorically (dichotomous [21,25,26], tertiles [18,24] and quartiles [19,48,50], with the lowest category being the reference group and referring to lowest consumption). The remaining three studies coded ultra-processed food consumption continuously [17,23,30]. 

Of the six studies that modelled mental disorder parameters as the exposure, one was coded continuously [22], with the remaining five coding these categorically (dichotomous [20,28,29], tertiles [27] and between-group comparison of patients with anorexia nervosa, bulimia nervosa and binge eating disorder [31]). 

Eleven studies reported average ultra-processed food consumption, and various methods were used, with some studies using more than one approach. These methods included energy per day [19,24,28,30,48,50]; grams per day [28,48,49]; the proportion of total food intake [23,31]; mean frequency of consumption score [17]; servings per day [50]; intake in the past week [27]; and grams per kilocalories [18] (see Table S2 for a detailed summary of the ultra-processed food exposure and outcome variables and average ultra-processed food consumption). However, the average intake of ultra-processed food was most commonly expressed as a percentage of total energy, which had a mean value of 32% and ranged from 17.3% to 54.9% [19,28,48,50]. 

### 3.4. Meta-Analyses and Narrative Syntheses

See Table 2 below for a summary of associations from our meta-analyses and from studies included only in the narrative syntheses. 

#### 3.4.1. Common Mental disorders

##### Meta-Analysis

**Figure 1:**
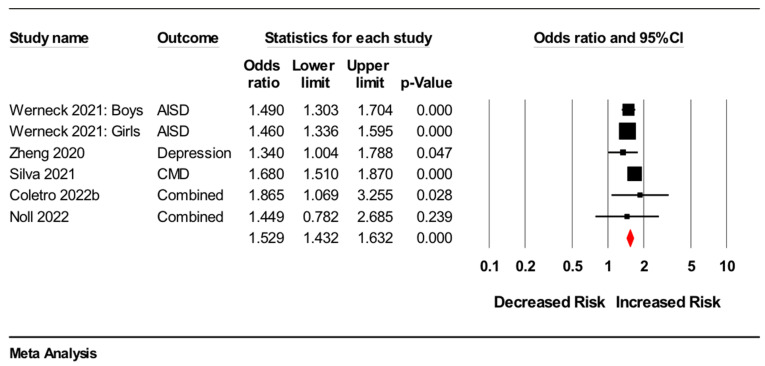
Forest plot of meta-analysis for cross-sectional studies assessing association between higher versus lower consumption of ultra-processed food and odds of common mental disorder symptoms. Note: AISD: anxiety-induced sleep disturbance; CMD: common mental disorders. For ‘Coletro 2022b’ and ‘Noll 2022′, effect estimates for depressive and anxiety symptoms were combined [18,19,21,24,25].

Five cross-sectional studies [18,19,21,24,25] were included in a meta-analysis that examined associations between ultra-processed food intake and symptoms of the common mental disorders, depression and anxiety (*n* = 185,773) (see Table 1 and Table S2 for study characteristics and variable details). Results showed that greater ultra-processed food consumption was associated with higher odds of depressive and anxiety symptoms (odds ratio: 1.53, 95%CI 1.43 to 1.63, *p* < 0.001, I^2^ = 8.9%) (see Figure 1 for forest plot). Two sensitivity analyses showed similar results to the primary analysis, with one including only adult participants (*n* = 15,555; odds ratio: 1.44, 95%CI 1.14 to 1.82, *p* = 0.003) and the other including only adolescent participants (*n* = 170,218; odds ratio: 1.53, 95%CI 1.44 to 1.63, *p* < 0.001); we also observed no differences between age-groups (*p* = 0.601). 

##### Narrative Synthesis

One further cross-sectional study examined associations between ultra-processed food consumption and internalising symptoms (e.g., problems of withdrawal, somatic complaints) in Brazilian adolescents (mean age of 15 years, *n* = 2680) [17]. This study reported an association between greater ultra-processed food consumption and higher levels of internalising symptoms (beta coefficient: 0.12, *p* < 0.001; 95%CI not reported) [17].

#### 3.4.2. Depression 

Eight studies [19,20,21,22,23,24,48,50] assessed associations between ultra-processed food intake and depression (*n* = 102,005). 

##### Meta-Analyses

Of these eight studies, two [48,50] were prospective by design and conducted in Spain (*n* = 14,907) and France (*n* = 26,730). These studies were included in our previously published meta-analysis that reported greater ultra-processed food intake was associated with an increased risk of incident depression diagnosis or depressive symptoms (hazard ratio: 1.22, 95%CI 1.16 to 1.28; *p* < 0.001; I^2^ = 0%, *n* = 41,637) (see Figure S6 in reference [16], also presented in Figure S3) [16]. 

**Figure 2:**
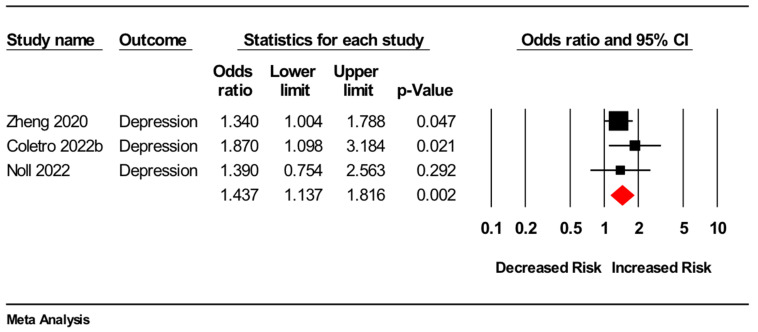
Forest plot of meta-analysis for cross-sectional studies assessing association between higher versus lower consumption of ultra-processed food and odds of depressive symptoms [19,21,24].

The other six studies were cross-sectional [19,20,21,22,23,24]. Three of these [19,21,24] were included in a meta-analysis (*n* = 15,555), which showed greater ultra-processed food consumption was associated with higher odds of depressive symptoms (odds ratio: 1.44, 95%CI 1.14 to 1.82, *p* = 0.002, I^2^ = 0%) (see Figure 2 for forest plot). Our main findings were unmodified by the one-study-removed sensitivity analyses (data not shown).

##### Narrative Syntheses

In the remaining three of six cross-sectional studies not included in the depressive symptoms meta-analysis, one was undertaken in patients diagnosed with alcohol use disorder and hospitalised for a three-week detoxification program (*n* = 48) [23]. This study reported little evidence of an association between ultra-processed food intake and depressive symptoms (Pearson correlation: 0.32, estimated 95%CI 0.00 to 0.52, *p* = 0.056) [23]. 

The other two cross-sectional studies were examined in the context of the COVID-19 pandemic [20,22]. One of these was conducted in Brazilian adults (*n* = 42,024) [20], and reported that participants with a previous diagnosis of depression were more likely to present with an elevated frequency of ultra-processed food consumption during COVID-19 confinement than participants without depression (odds ratio: 1.49, 95%CI 1.21 to 1.83, estimated *p* < 0.001) [20]. 

The second COVID-19 study was conducted in Italy across two separate samples [22]. Using data from the first sample (*n* = 1340), higher levels of depressive symptoms were associated with greater changes to ultra-processed food consumption during COVID-19 confinement (beta coefficient for Patient Health Questionnaire-9: 0.16, 95%CI 0.10 to 0.22 and Screening Questionnaire for Disaster Mental Health: 0.17, 95%CI 0.11 to 0.23, respectively). Similar associations were observed in the second sample (*n* = 1401): higher levels of depressive symptoms were associated with greater changes to ultra-processed food consumption during COVID-19 confinement (beta coefficient for Patient Health Questionnaire-9: 0.07, 95%CI 0.02 to 0.13, *p* < 0.0001 and Screening Questionnaire for Disaster Mental Health: 0.13, 95%CI 0.08 to 0.18, *p* < 0.0001, respectively).

#### 3.4.3. Anxiety

Six cross-sectional studies [21,22,23,24,25,26] assessed associations between ultra-processed food intake and anxiety symptoms (*n* = 205,146), including anxiety-induced sleep disturbance [25,26]. 

##### Meta-Analysis

**Figure 3:**
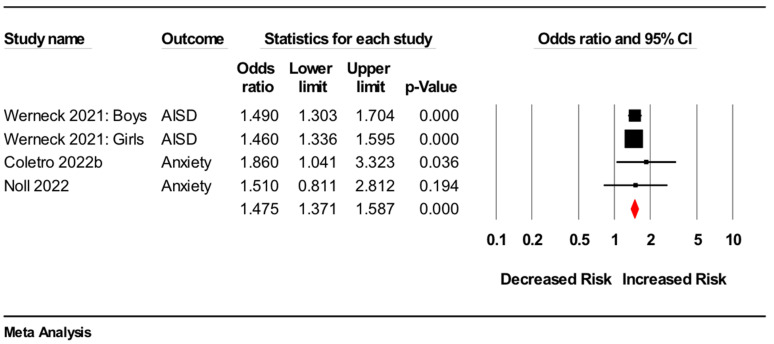
Forest plot of meta-analysis for cross-sectional studies assessing associations between higher versus lower consumption of ultra-processed food and odds of anxiety symptoms [21,24,25]. Note: AISD: anxiety-induced sleep disturbance.

Of these six studies, three [21,24,26] were included in a meta-analysis (*n* = 101,709). This meta-analysis showed that greater ultra-processed food consumption was associated with higher odds of anxiety symptoms (odds ratio: 1.48, 95%CI 1.37 to 1.59, *p* < 0.001, I^2^ = 0%) (see Figure 3 for forest plot). These results were consistent with the results from sensitivity analyses (data not shown).

##### Narrative Syntheses 

Of the three remaining studies not included in the anxiety symptoms meta-analysis, one was undertaken in patients diagnosed with alcohol use disorder and hospitalised for a three-week detoxification program (*n* = 48) [23]. There was little evidence of an association between ultra-processed food intake and anxiety symptoms (Pearson correlation: 0.24, estimated 95%CI -0.05 to 0.49, *p* = 0.10) [23]. 

One study was undertaken in the context of the COVID-19 pandemic [22]. This study, conducted in Italy, examined associations between anxiety symptoms and ultra-processed food intake across two separate samples [22]. Using data from the first (1340) and second samples (*n* = 1401), higher levels of anxiety symptoms were associated with greater changes to ultra-processed food consumption during COVID-19 confinement (beta coefficients: 0.14, 95%CI 0.08 to 0.20, *p* < 0.0001 and 0.08, 95%CI 0.02 to 0.13, *p* = 0.01, respectively). 

The final anxiety-related study used an exposure that combined high ultra-processed food intake with high sedentary behaviour to assess the simultaneous impact of both on anxiety-induced sleep disturbance in Brazilian adolescents (*n* = 100,648) [26]. This study reported high ultra-processed food intake in conjunction with high sitting time was associated with increased odds of anxiety-induced sleep disturbance (odds ratio for boys: 1.85, 95%CI 1.46 to 2.35, estimated *p* < 0.001 and girls: 1.62, 95%CI 1.39 to 1.89, estimated *p* < 0.001). In addition, high ultra-processed food intake in conjunction with high television viewing was associated with increased odds of anxiety-induced sleep disturbance (odds ratio for boys: 2.03, 95%CI 1.61 to 2.56, estimated *p* < 0.001 and girls: 2.04, 95%CI 1.76 to 2.36, estimated *p* < 0.001) [26].

#### 3.4.4. Trauma and Stress

##### Narrative Syntheses

One cross-sectional study conducted in Italy examined associations between post-traumatic stress disorder symptoms and ultra-processed food intake in the context of COVID-19 across two separate samples [22]. Using data from the first (*n* = 1340) and second samples (*n* = 1401), higher levels of post-traumatic stress disorder symptoms were associated with greater changes to ultra-processed food consumption during COVID-19 confinement (beta coefficients: 0.10, 95%CI 0.04 to 0.16, *p* = 0.001 and 0.09, 95%CI 0.03 to 0.14, *p* = 0.001, respectively) [22].

Three cross-sectional studies [22,27,28] examined associations between perceived stress levels and ultra-processed food consumption (*n* = 12,580). 

One of these assessed associations in young Brazilian adults (*n* = 1270) working in the industrial and retail sectors [27]. This study reported high perceived stress was associated with elevated odds of greater ultra-processed food consumption (odds ratio: 1.94, 95%CI 1.54 to 2.45, estimated *p* < 0.001) [27]. 

The other two studies were conducted in Italy. One of these examined associations in the context of COVID-19 confinement across two separate samples [22]. Using data from the first sample (*n* = 1340), higher levels of perceived stress were associated with greater changes to ultra-processed food consumption during COVID-19 confinement (beta coefficient: 0.10, 95%CI 0.04 to 0.16, *p* = 0.001). In contrast, results from the second sample (*n* = 1401) showed no evidence of an association (beta coefficient: −0.04, 95%CI −0.09 to 0.01, *p* = 0.15) [22].

The other study in Italian adults (*n* = 8569) reported stress at work sometimes/most of the time versus no stress was inversely associated with ultra-processed food consumption (beta coefficient: −2.98, 95%CI −4.28 to −1.13, *p* = 0.0016) [28]. Stress at home sometimes (beta coefficient: -3.05, 95%CI -4.62 to -1.48, *p* = 0.0001) and stress at home most of the time (beta coefficient: −2.95, 95%CI −4.54 to −1.37) were also inversely associated with ultra-processed food consumption. However, there was little evidence for associations of stress at work often/always (beta coefficient: −1.17, 95%CI −3.28 to 0.95, *p* = 0.28) and stress at home often/always (beta coefficient: 0.55, 95%CI −1.52 to 2.61) with ultra-processed food consumption [28]. 

#### 3.4.5. Addiction

##### Narrative Syntheses 

Three cross-sectional studies [23,29,30] assessed the link between ultra-processed food consumption and addiction (*n* = 126), such as food addiction diagnosis [29,30] and alcohol use disorder symptoms [23]. 

One of these was conducted in Brazilian children with high body mass index for age (defined as ≥1 Z score, mean age of 10 years, *n* = 33) [30]. This study reported that consumption of certain ultra-processed foods such as cookies and savoury biscuits was associated with food addiction diagnoses (odds ratio: 4.19, 95%CI 1.32 to 13.26, *p* = 0.025) [30]. However, evidence of an association between the intake of sausages and food addiction was less certain (odds ratio: 11.77, 95%CI 1.29 to 107.42, *p* = 0.05), given the very wide confidence interval [30]. 

A separate study in American adults (*n* = 45) reported that a food addiction diagnosis was associated with greater intake of ultra-processed food before (beta coefficient: 1.08, estimated 95%CI 0.69 to 1.47, *p <* 0.001) and during COVID-19 (beta coefficient: 1.18, estimated 95%CI 0.81 to 1.55, *p <* 0.001) [29]. 

The last study was undertaken in patients diagnosed with alcohol use disorder and hospitalised for a three-week detoxification program (*n* = 48) [23]. Greater ultra-processed food intake was moderately associated with alcohol use disorder symptoms, such as obsessive alcohol cravings (Pearson correlation: 0.32, estimated 95%CI 0.04 to 0.55, *p* = 0.03), but was not correlated with compulsive alcohol cravings (Pearson correlation: 0.13, estimated 95%CI -0.16 to 0.40, *p* = 0.39) [23].

#### 3.4.6. Eating Disorders

##### Narrative Synthesis

One cross-sectional retrospective study [31] investigated the link between disordered eating in a clinical sample/setting and ultra-processed food consumption (*n* = 73). Patients diagnosed with anorexia nervosa (*n* = 22) reported ultra-processed food contributed 55% of their daily dietary intake in the two weeks prior to measurement, whereas patients with binge eating disorder (*n* = 26) and bulimia nervosa (*n* = 25) reported that ultra-processed food contributed 69–72% of their daily dietary intake. However, there was little evidence of a between-group difference (Chi-squared test: *p* = 0.19) [31]. In addition, all foods consumed in a binge eating pattern were ultra-processed [31]. Artificially sweetened beverages and low-fat products were common ultra-processed food items [31].

## 4. Discussion

We present a systematic review and meta-analysis of 385,541 participants that reports associations between ultra-processed food consumption and a range of mental disorder parameters. Results from a series of meta-analyses involving cross-sectional studies demonstrated that greater intake of ultra-processed food was associated with increased odds of depressive and anxiety symptoms, both when these outcomes were assessed together as well as separately. Furthermore, in a meta-analysis of prospective studies, greater ultra-processed food intake was associated with an increased risk of subsequent depressive outcomes. Our narrative syntheses of studies not eligible for meta-analysis showed that for 65% of analyses, intake of ultra-processed food was positively and cross-sectionally associated with depressive, anxiety, trauma and stress as well as addiction-related parameters. These findings build upon the extensive body of evidence that demonstrates healthier dietary patterns characterised by higher intakes of fruit, vegetables, whole grains, fish, olive oil and low-fat dairy, and lower levels of ultra-processed food, such as the Mediterranean and ‘anti-inflammatory’ diets [5,6,8,51,52,53], are associated with reduced risk of mental disorders such as depression.

While the majority of studies included in our review used a cross-sectional design (88%), where inferences regarding the direction of associations are limited, ultra-processed food consumption appeared to be bidirectionally associated with adverse mental health. Nonetheless, all of the studies included in our meta-analyses, and 65% included in our narrative syntheses, modelled and demonstrated direct associations between ultra-processed food consumption as the exposure variable and mental disorder parameters as the outcome. Numerous hypotheses support this implied causal pathway. Although robust evidence is currently limited [54], a well-established and consistent body of literature demonstrates that, although NOVA largely ignores the nutritional composition of food in its classification process, many ultra-processed foods are sources of high energy, refined starches, sugar, sodium and saturated and trans-fats [11]. Ultra-processed foods also typically lack the various fibres, polyphenols, omega-3 fatty acids and essential vitamins and minerals of non-ultra-processed foods such as vegetables, fruits, legumes, wholegrains, fatty fish, lean meats, nuts and seeds and others [11]. These nutrient-poor profiles have been implicated in the prevalence, incidence and severity of depression through a number of interacting pathways, including inflammation, oxidative stress and the gut microbiome [6]. 

Part of the association between ultra-processed consumption and mental disorders may also be explained by non-nutritive components used or produced via food ultra-processing. Multiple non-nutritive components of ultra-processed foods have been implicated in the modulation of pathways relevant to mental disorders. Limited but supporting data suggest that greater intakes of artificial sweeteners (aspartame, saccharin) and monosodium glutamate (MSG) may be involved in dysregulating the synthesis and release of neurotransmitters implicated in mood disorders, such as dopamine, norepinephrine and serotonin [55] in addition to the hypothalamic-pituitary adrenal (HPA) axis [56]. Preclinical and clinical studies suggest a possible role for the emulsifiers carboxymethylcellulose [57,58] and polysorbate-80 [58,59], used as antimicrobial agents, in the link between ultra-processed food and mental disorders. These compounds may alter the gut microbiota composition (reduced diversity) and function (reduced short-chain fatty acids and free amino acids) and foster associated inflammatory responses [60,61]. Ingestion of titanium dioxide nanoparticles, widely used for their whiteness and opacity as food colourants, has been linked to higher concentration of the inflammatory cytokine interleukin-6 in the plasma and cerebral cortex and associated neuroinflammation in rats [62]. Indeed, inflammation is implicated in influencing or predicting the prevalence, incidence and treatment response of mental disorders, and peripheral measurements of inflammation have also been proposed as plausible biomarkers of mental disorders [63]. The relevance of inflammation as a possible mediator on the causal pathway from ultra-processed consumption food to mental disorders warrants further investigation. Preclinical studies also demonstrate that exposure to titanium dioxide nanoparticles may cause the destruction of dopaminergic neurons [64]. In addition, a recent review suggested that perinatal exposure to Bisphenol A, a compound used in the production of plastic food and drink containers and packages, disrupts stress-sensitive and endocrine systems that may translate to anxious and depressive states later in life [65]. 

Common mental disorders such as depression are often comorbid and share a bidirectional relationship with higher body mass index [66,67]. Other aspects of ultra-processed food may be indirectly associated with depression via their impact on adiposity. These include the potential for ultra-processed food to: (1) be relatively high energy density compared to non-ultra-processed food [54]; (2) foster overconsumption and subsequent excess energy intake due to its sensory attributes [68]; and (3) alter gut-brain signalling or flavour-nutrient feedback loops [69]. However, the role of adiposity in the association between ultra-processed food and mental disorders is likely to be highly complex, particularly in the context of eating disorders. Our results did not shed light on this due to an absence of data and limited analytical approaches, which showed little evidence of a between-group difference in consumption of ultra-processed food across patients diagnosed with different eating disorders. 

The causal pathway implied by 35% of the studies included in our narrative syntheses [20,22,27,28,29,31], which modelled mental disorder parameters as the exposure and ultra-processed food consumption as the outcome, may partly be explained by the HPA axis. Due to phenomena known as emotional eating and comfort food, both chronic and acute uncontrolled stress can dysregulate the HPA axis, which may in turn influence multiple appetite-related hormones (noradrenaline and cortisol) and hypothalamic neuropeptides (corticotropin-releasing factor) [70]. Hyperactivity of the HPA axis may also alter eating behaviour by heightening the preference for and overconsumption of hyper-palatable and energy-dense foods [70], such as those that are ultra-processed. This is particularly relevant for the COVID-19 context-specific associations reported in our review [20,21,22,29], and suggests individuals might turn to ultra-processed food in an attempt to mitigate stress-related anxiety based on events outside of their control [71]. Adverse mental health as a risk factor for greater ultra-processed food consumption may have consequences for other metabolic outcomes by which experimental (randomised controlled trial) and epidemiological (prospective) evidence has implicated elevated ultra-processed food consumption, including weight gain [72] or higher body mass index [73]. More mechanistic studies in humans are necessary to tease apart the precise attributes of ultra-processed food that confer harm and elucidate whether the observed bidirectional associations between consumption of ultra-processed food and mental or related physical health are causal. 

### 4.1. Limitations and Future Directions

Consideration of the following limitations is recommended when interpreting our results. As previously mentioned, the majority of included studies used a cross-sectional design (88%). More prospective research with repeated measurements of diet is required to better investigate the role of ultra-processed food in habitual dietary intake and mental disorders, especially in terms of directionality and temporality. Similarly, while the majority of studies included in our systematic review adjusted for potential covariates, observational studies will always run the risk of residual confounding by many factors. To demonstrate causality, intervention studies are needed; however, these are unlikely to be conducted given the likely noxious impact of these foods on health outcomes.

Between-study differences in analytical approaches were also noted, which included ultra-processed food intake modelled as either the exposure or outcome variable and coded both continuously and categorically (with different cut-offs) (see Table S2 for variable characteristics, and the results section “Details of exposure variables and average ultra-processed food consumption” for a description of the different approaches). This makes it challenging to directly compare some studies and estimate how much ultra-processed food intake is required to potentially confer adverse mental health. We encourage standardised analytical approaches that consider the information presented in our review. For example, previous studies [74,75,76,77] have sought to better account for ultra-processed foods that provide little to no energy (e.g., artificially sweetened beverages) by using the weight of ultra-processed food in grams per day adjusted for energy. Only two studies included in our review used the weight of ultra-processed food as the exposure variable [48,49] (see Table S2 for a detailed summary of the exposure variables). In addition, although we used well established and previously reported methods [45,46] to estimate the confidence intervals for two studies [23,29], these methods are not without limitations [45]. Future studies are encouraged to report confidence intervals for their estimates of effect. Furthermore, a considerable proportion of studies included in our systematic review (53%) and meta-analyses (80%) were conducted in one region, namely Brazil [17,18,20,21,24,25,26,27,30]. Replicating these findings in other regions is needed to allow for greater generalisability.

Our critical appraisal process noted that it was unclear whether strategies to deal with confounding factors were adequate in three studies [23,31] (see Tables S3 and S4 for risk of bias assessment). In addition, for several studies, it was unclear whether the dietary tools used were validated to capture habitual dietary intake (58%) [18,20,21,22,25,26,27,28,29,31] or whether mental disorder parameters were measured using a valid and reliable method (24%) [20,25,26,28]. Due to the small number of studies included in our meta-analyses (*n* ≤ 5), we were unable to conduct sensitivity analyses or meta-regressions to explore these limitations as possible sources of heterogeneity. Relatedly, to date, there are currently no available dietary tools that are validated to capture ultra-processed food consumption. Twenty-four-hour dietary recalls, which were utilised by 35% of the studies in our review [18,19,23,24,28,48], allow for the inclusion of any and all reported food items, and may better capture food intake by the degree of processing compared to food frequency questionnaires that contain predefined lists of food items [78]. Indeed, twenty-four-hour dietary recalls and food frequency questionnaires are different in practice. Research also shows that three repeated twenty-four-hour dietary recalls are needed to be accurately representative of habitual dietary intake, with three repeated measures reducing measurement error compared to one or two [79]. Three of six studies included in our review used singular twenty-four-hour dietary recalls [18,19,28] and future studies are encouraged to address this shortfall. The field would benefit from the development of tools specifically designed to assess ultra-processed food consumption. 

### 4.2. Implications

The past several decades have seen an overrepresentation in the manufacturing, sales and intake of ultra-processed foods for many food systems globally, with future projections showing a continued upward trend [13,14]. The precautionary principle to address ultra-processed food consumption (as well as production and distribution) in new official dietary guidelines developed by governmental and international health organisations is being called for [80] and increasingly adopted [81]. This recommendation is supported by the growing body of evidence showing ultra-processed food consumption contributes to suboptimal mental as well as physical health and mortality [16]. Notwithstanding the noted limitations, our review adds to this evidence and reinforces existing observational and experimental data demonstrating a role for healthier dietary patterns [6,8,51,52,82] and adjunctive dietary interventions [83,84,85,86], such as Mediterranean and ‘anti-inflammatory’ diets, in the prevention and treatment of mental disorders.

## 5. Conclusions

The present review suggests bidirectional associations exist between the intake of ultra-processed food and adverse mental health. The strongest evidence was derived from meta-analyses largely consisting of cross-sectional studies that modelled ultra-processed food consumption as the exposure variable and symptoms of the common mental disorders, depression and anxiety, as the outcome. These meta-analyses demonstrated direct associations, both when depressive and anxiety symptoms were assessed together as well as separately. Further rigorously designed prospective and experimental studies are needed to better determine directionality and causality and ensure that global preventative and treatment strategies are efficacious and appropriate.

## Figures and Tables

**Table 1:** Study characteristics and key findings.

Author/Year	Study Characteristics	Confounding Variables	Mental Disorder Parameters	Results	Overall Critical Appraisal
Adjibade et al., 2019 [48]	**Study design:** Prospective **Sample size:** 26,730 **Country:** France **Population:** Adults **Dietary assessment:** 3 × 24-h dietary records	Age, sex, body mass index, marital status, educational level, occupational categories, household income per consumption unit, residential area, number of 24-h dietary records, inclusion month, energy consumption without alcohol, alcohol consumption, smoking status, and physical activity	- Depressive symptoms	↑ vs. ↓ UPF = ↑ Depressive symptoms (hazard ratio for 10% increase in ultra-processed food 1.21, 95%CI 1.15 to 1.27, *p* < 0.0001)	No concerns.
Amadieu et al., 2021 [23]	**Study design:** Cross sectional **Sample size:** 48 **Country:** Belgium **Population:** Adults **Dietary assessment:** 3 × 24-h dietary records	Total energy intake	- Depression - Anxiety - Alcohol craving	↑ vs. ↓ UPF ≠ Depression (Pearson correlation: 0.32, estimated 95%CI 0.00 to 0.52, *p* = 0.056) ≠ Anxiety (Pearson correlation: 0.24, estimated 95%CI -0.05 to 0.49, *p* = 0.10) ≠ Compulsive alcohol craving (Pearson correlation: 0.13, estimated 95%CI −0.16 to 0.40, *p* = 0.39) = ↑ Obsessive alcohol craving (Pearson correlation: 0.32, estimated 95%CI 0.04 to 0.55, *p* = 0.03)	Potential bias: strategies to deal with confounding factors and statistical analysis domains.
Ayton et al., 2021 [31]	**Study design:** Cross sectional **Sample size:** 73 Country: UK **Population:** Adults and adolescents **Dietary assessment:** Clinician documented dietary intake by asking the patient to describe “a typical food intake per day over the past 2 weeks”	None	- Anorexia Nervosa - Bulimia Nervosa - Binge Eating Disorder	No between-group difference in average ultra-processed food consumption (Chi-squared test: *p* = 0.19): Patients with Anorexia Nervosa = 55% Patients with Bulimia Nervosa = 72% Patients with Binge Eating Disorder = 69% - Foods that were consumed in a binge eating pattern were 100% ultra-processed.	Potential bias: inclusion criteria; measurement validity; strategies to deal with confounding factors and statistical analysis domains.
Bonaccio et al., 2021 [22]	**Study design:** Cross sectional **Sample size:** 2741 **Country:** Italy **Population:** Adults **Dietary assessment:** Food-frequency questionnaire	Age, sex, geographical area, living area, educational level, household income, marital status, number of cohabitants, occupational class, history of chronic diseases, diagnosis of ≥1 disease during confinement, use of psychoactive drugs before and during lockdown	- Depression - Anxiety - Stress - Post-Traumatic Stress Disorder	↑ vs. ↓ UPF RISCOVID-19 sample = ↑ Depression (beta coefficient for Patient Health Questionnaire-9: 0.16, 95%CI 0.10 to 0.22, *p* < 0.0001 and Screening Questionnaire for Disaster Mental Health: 0.17, 95%CI 0.11 to 0.23, *p* < 0.0001, respectively) = ↑ Anxiety (beta coefficient: 0.14, 95%CI 0.08 to 0.20, *p* < 0.0001) = ↑ Stress (beta coefficient: 0.10, 95%CI 0.04 to 0.16, *p* = 0.001) = ↑ Post-Traumatic Stress Disorder (beta coefficient: 0.10, 95%CI 0.04 to 0.16, *p* = 0.001) Moli-LOCK sample = ↑ Depression (beta coefficient for Patient Health Questionnaire-9: 0.07, 95%CI 0.02 to 0.13 and Screening Questionnaire for Disaster Mental Health: 0.13, 95%CI 0.08 to 0.18) = ↑ Anxiety (beta coefficient: 0.08, 95%CI 0.02 to 0.13, *p* = 0.01) ≠ Stress (beta coefficient: −0.04, 95%CI −0.09 to 0.01, *p* = 0.15) = ↑ Post-Traumatic Stress Disorder (0.09, 95%CI 0.03 to 0.14, *p* = 0.001)	Potential bias: measurement validity domain.
Coletro et al., 2021 [21]	**Study design:** Cross sectional **Sample size:** 1693 **Country:** Brazil **Population:** Adults **Dietary assessment:** Food-frequency questionnaire	Sex, age, marital status, educational background, family income and medical diagnosis of depression or anxiety disorders	- Depression - Anxiety	↑ vs. ↓ UPF = ↑ Depressive symptoms (odds ratio: 1.87, 95%CI 1.10 to 3.19, *p* = 0.022) ↑ Anxiety symptoms (odds ratio: 1.86, 95%CI 1.04 to 3.32, *p* = 0.036)	Potential bias: measurement validity domain.
Faisal-Cury et al., 2021 [17]	**Study design:** Cross sectional **Sample size:** 2680 **Country:** Brazil **Population:** Adolescents **Dietary assessment:** Food-frequency questionnaire	Sex, age, skin colour, indigenous mother schooling, school administrative dependency, physical activity practice and the habit of having meals with parents	- Internalising Symptoms	↑ vs. ↓ UPF = ↑ Internalising Symptoms (beta coefficient: 0.12, *p* < 0.001; 95%CI not reported)	No concerns.
Filgueiras et al., 2019 [30]	**Study design:** Cross sectional **Sample size:** 33 **Country:** Brazil **Population:** Children **Dietary assessment:** Semi-quantitative food-frequency questionnaire	Sugar, salt and fat consumption	- Food addiction	Food addiction vs. no food addiction = ↑ Cookies/biscuits intake (odds ratio: 4.19, 95%CI 1.32 to 13.26, *p* = 0.025) = ↑ Sausages intake (odds ratio: 11.77, 95%CI 1.29 to 107.42, *p* = 0.05)	Potential bias: strategies to deal with confounding factors domain.
Gómez-Donoso et al., 2019 [49]	**Study design:** Prospective **Sample size:** 14,907 **Country:** Spain **Population:** Adults **Dietary assessment:** Semi-quantitative food-frequency questionnaire	Sex, stratified by age groups, and year of entrance to the cohort, baseline BMI, total energy consumption, physical activity, smoking status, marital status, living alone, employment status, working hours per week, health-related career, years of education, adherence to Trichopoulou’s MeDiet Score, and baseline self-perception of competitiveness, anxiety and dependence levels	- Depression	↑ vs. ↓ UPF = ↑ Depression (hazard ratio: 1.33, 95%CI 1.07 to 1.64, *p* trend = 0.004)	No concerns.
Lopes Cortes et al., 2021 [27]	**Study design:** Cross sectional **Sample size:** 1270 **Country:** Brazil **Population:** Adults **Dietary assessment:** Food-frequency questionnaire	Sex, age, educational level, socioeconomic status, marital status, smoking, high-risk alcohol consumption, physical activity status, BMI status, and self-rated health	- Stress	High vs. low/moderate perceived stress = ↑ UPF consumption (odds ratio: 1.94, 95%CI 1.54 to 2.45, estimated *p* < 0.001)	Potential bias: measurement validity domain.
Noll et al., 2022 [24]	**Study design:** Cross sectional **Sample size:** 225 **Country:** Brazil **Population:** Adults **Dietary assessment:** 3 × 24-h dietary records	Age, marital status, income, and early and late post-menopause	- Depression - Anxiety	↑ vs. ↓ UPF ≠ Depression (odds ratio: 1.39, 95%CI 0.75 to 2.55, *p* = 0.292) ≠ Anxiety (odds ratio: 1.51, 95%CI 0.81 to 2.81, *p =* 0.195)	No concerns.
Ruggiero et al., 2020 [28]	**Study design:** Cross sectional **Sample size:** 8569 **Country:** Brazil **Population:** Adults **Dietary assessment:** 1 × 24-h dietary record	Age, sex and energy intake, education, geographical area, place of residence, sport activity, occupation, marital status, smoking, BMI, CVD, cancer, hypertension, diabetes and hyperlipidaemia	- Stress at work - Stress at home	- Stress at work sometimes/most times vs. no stress = ↓ UPF (beta coefficient: −2.98, 95%CI −4.28 to −1.13, *p* = 0.0016) - Stress at work often/always vs. no stress ≠ UPF (beta coefficient: −1.17, 95%CI −3.28 to 0.95, *p* = 0.28) - Stress at home sometimes vs. no stress = ↓ UPF (beta coefficient: −3.05, 95%CI −4.62 to −1.48, *p* = 0.0001) - Stress at home often/always vs. no stress ≠ UPF (beta coefficient: 0.55, 95%CI −1.52 to 2.61, *p* = 0.60)	Potential bias: measurement validity domain.
Schulte et al., 2022 [29]	**Study design:** Cross sectional **Sample size:** 45 **Country:** USA **Population:** Adults **Dietary assessment:** Food-frequency questionnaire	Height and weight measurements considered biologically implausible values (height <44 inches (112 cm) or >90 inches (229 cm); weight <55 lb (24.95 kg) or >1000 lb (453.59 kg)), incorrectly answering “catch questions,” which have commonly-known answers (e.g., 2 + 2) designed to “catch” participants who respond without reading the questions carefully	- Food addiction	Food addiction vs. no food addiction = ↑ UPF before (beta coefficient: 1.08, estimated 95%CI 0.69 to 1.47, *p <* 0.001) and during COVID-19 (beta coefficient: 1.18, estimated 95%CI 0.81 to 1.55, *p <* 0.001)	Potential bias: measurement validity domain.
Silva et al., 2021 [18]	**Study design:** Cross sectional **Sample size:** 70,427 **Country:** Brazil **Population:** Adolescents **Dietary assessment:** 1 × 24-h dietary record	Chronological age, ethnicity, region of the country, type of city (capital or interior), and physical activity	- Common Mental Disorders	↑ vs. ↓ UPF = ↑ Common Mental Disorders (odds ratio: 1.68; 95% CI 1.51 to 1.87, *p <* 0.001)	Potential bias: measurement validity domain.
Werneck et al., 2020 [26]	**Study design:** Cross sectional **Sample size:** 100,648 **Country:** Brazil **Population:** Adults **Dietary assessment:** Food-frequency questionnaire	Chronological age, ethnicity, region of the country, type of city (capital or interior), and physical activity	- Anxiety-Induced Sleep Disturbance	↑ vs. ↓ UPF with high sedentary behaviour = ↑ Anxiety-Induced Sleep Disturbance (odds ratio for boys: 1.85, 95%CI 1.46 to 2.35, estimated *p* < 0.001 and girls: 1.62, 95%CI 1.39 to 1.89, estimated *p* < 0.001) ↑ vs. ↓ UPF with high television viewing = ↑ Anxiety-Induced Sleep Disturbance (odds ratio for boys: 2.03, 95%CI 1.61 to 2.56, estimated *p* < 0.001 and girls: 2.04, 95%CI 1.76 to 2.36, estimated *p* < 0.001)	Potential bias: inclusion criteria and measurement validity domains.
Werneck et al., 2020 COVID [20]	**Study design:** Cross sectional **Sample size:** 42,024 **Country:** Brazil **Population:** Adolescents **Dietary assessment:** Food-frequency questionnaire	Sex, age group, highest academic achievement, working status during the pandemic, skin colour, alcohol use, tobacco smoking, diagnoses of COVID-19 on a close friend, co-worker or relative and adherence to the quarantine	- Depression	Depression vs. no depression = ↑ UPF consumption incidence (odds ratio: 1.49, 95%CI 1.21 to 1.83, estimated *p* < 0.001)	Potential bias: inclusion criteria and measurement validity domains.
Werneck et al., 2021 [25]	**Study design:** Cross sectional **Sample size:** 99,791 **Country:** Brazil **Population:** Adolescents **Dietary assessment:** Food-frequency questionnaire	Age group, ethnicity, food insecurity, country region, type of city and physical activity	- Anxiety-Induced Sleep Disturbance	↑ vs. ↓ UPF = ↑ Anxiety-Induced Sleep Disturbance (odds ratio for boys: 1.48, 95% CI: 1.3 to 1.7, estimated *p* < 0.001 and girls: 1.46, 95% CI: 1.34 to 1.6, estimated *p <* 0.001)	Potential bias: inclusion criteria and measurement validity domains.
Zheng et al., 2020 [19]	**Study design:** Cross sectional **Sample size:** 13,637 **Country:** USA **Population:** Adults **Dietary assessment:** 1 × 24-h dietary record	Age, sex, race, BMI, educational level, annual family income, marital status, physical activity, drinking, smoking, current hypertension, diabetes history, heart disease history, and chronic bronchitis.	- Depressive Symptoms	↑ vs. ↓ UPF = ↑ Depressive Symptoms (odds ratio: 1.34, 95%CI CI 1.00 to 1.78, *p* = 0.03)	Potential bias: measurement validity domain.

Note: ↑: higher, ↓: lower, ≠: no association, UPF: ultra-processed food consumption; ‘estimated 95%CI’ calculated using the estimated effects and *p*-values as per the methods proposed by Altman and Bland (2011) for beta-coefficients [45] and Bishara and Hittner (2017) for Pearson correlation coefficients [46]; ‘estimated *p*…’ calculated using the confidence intervals as per the methods proposed by Altman and Bland (2011) [47].

**Table 2:** Number and direction of associations from our meta-analyses (top part of table) and from studies included only in the narrative syntheses (bottom part of table).

Mental Disorder Parameters	Direct Association	Inverse Association	No Association
**Meta-analyses (MA)**			
Common mental disorders combined	**1** Cross-sectional MA: OR 1.53, 95%CI 1.43 to 1.63, *p* < 0.001, *N* = 185,773		
Depression	**2** (a) Prospective MA: HR 1.22, 95%CI 1.16 to 1.28; *p* < 0.001, *N* = 41,637 (b) Cross-sectional MA: OR 1.44, 95%CI 1.14 to 1.82, *p* = 0.002, *N* = 15,555		
Anxiety	**1** Cross-sectional MA: OR 1.48, 95%CI 1.37 to 1.59, *p* < 0.001, *N* = 101,709		
**Narrative synthesis of individual studies**			
Common mental disorders combined	**1** (Faisal-Cury, Leite et al., 2021) [17]		
Depression	**3** (Werneck, Silva et al., 2020, Bonaccio, Costanzo et al., 2021) [20,22]		**1** (Amadieu, Leclercq et al., 2021) [23]
Anxiety	**4** (Werneck, Vancampfort et al., 2020, Bonaccio, Costanzo et al., 2021) [22,26]		**1** (Amadieu, Leclercq et al., 2021) [23]
Trauma and stress	**4** (Bonaccio, Costanzo et al., 2021, Lopes Cortes, Andrade Louzado et al., 2021) [22,27]	**3** (Ruggiero, Esposito et al., 2021) [28]	**3** (Bonaccio, Costanzo et al., 2021, Ruggiero, Esposito et al., 2021) [22,28]
Addiction	**5** (Filgueiras, Pires de Almeida et al., 2019, Amadieu, Leclercq et al., 2021, Schulte, Kral et al., 2021) [23,29,30]		**1** (Amadieu, Leclercq et al., 2021) [23]

OR: odds ratio; HR: hazard ratio.

## Supplementary Materials

The following supporting information can be downloaded at: https://www.mdpi.com/article/10.3390/nu14132568/s1, Figure S1: PRIMSA flowchart; Figure S2: Forest plot of prospective meta-analysis for consumption of ultra-processed food and risk of depression; Table S1: Search terms across databases; Table S2: Summary of the exposure variables and average ultra-processed food consumption; Table S3: Critical Appraisal Checklist for Cross-Sectional Studies; Table S4: Critical Appraisal Checklist for Cohort Studies.
