# Supplementary material for: Ultra-Processed Food Consumption and Mental Health: A Systematic Review and Meta-Analysis of Observational Studies
Source: Nutrients. 2022 Jun 21;14(13):2568. doi: 10.3390/nu14132568 (PMC9268228; doi:10.3390/nu14132568)
Supplement: Supplementary file 1 [file nutrients-14-02568-s001.zip › nutrients-1753671-supplementary.pdf]

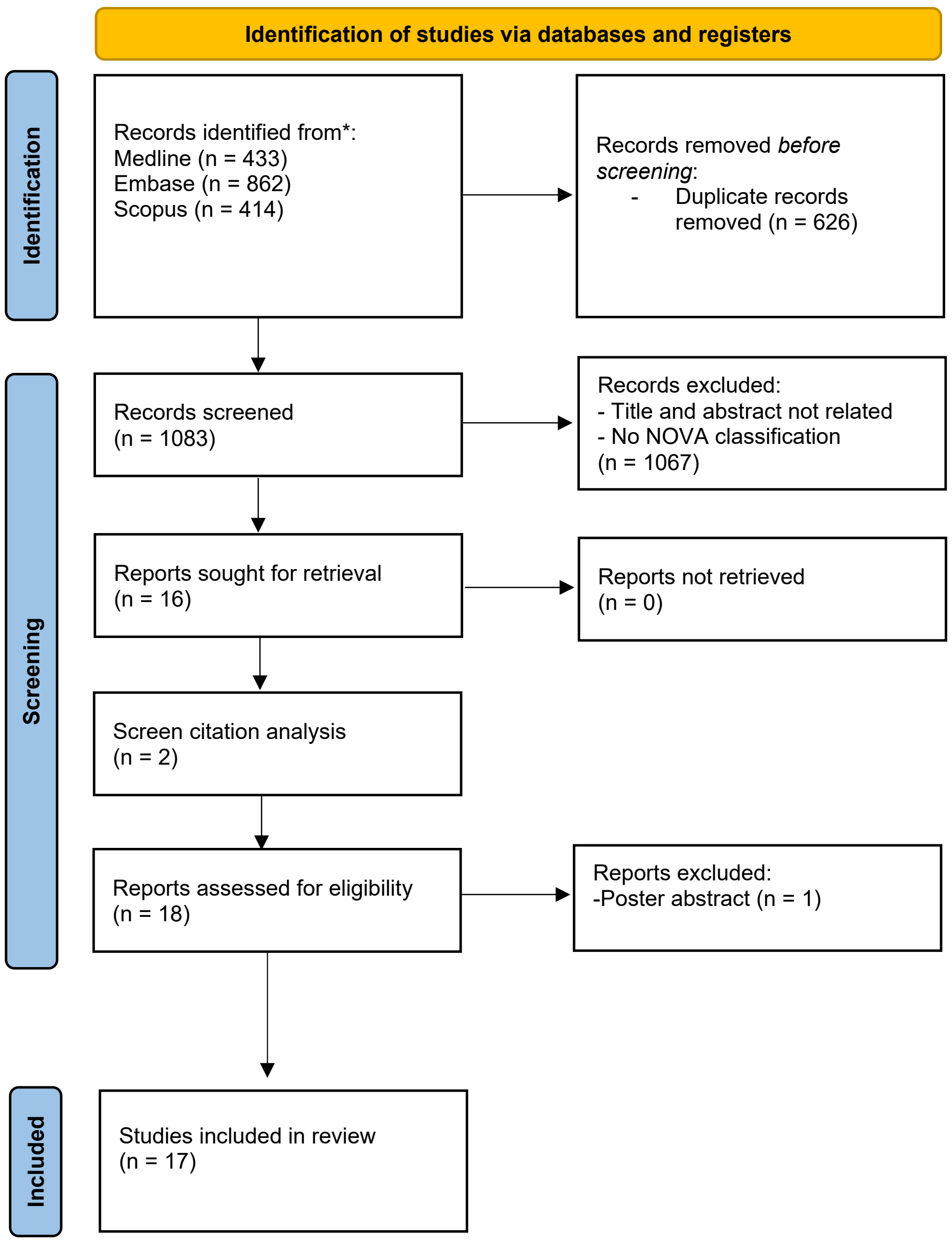

From: Page MJ, McKenzie JE, Bossuyt PM, Boutron I, Hoffmann TC, Mulrow CD, et al. The PRISMA 2020 statement: an updated guideline for reporting systematic reviews. BMJ 2021;372:n71. doi: 10.1136/bmj.n71

For more information, visit: http://www.prisma-statement.org/

Figure S1. PRISMA 2020 flow diagram for new systematic reviews which included searches of databases and registers only

Study name	Outcome	Statistics for each study				
		Hazard ratio	Lower limit	Upper limit	Z-Value	p-Value
Gómez-Donoso 2019	Depression	1.330	1.074	1.647	2.618	0.009
Adjibade 2019	Depression	1.210	1.151	1.272	7.528	0.000
		1.216	1.159	1.276	7.925	0.000

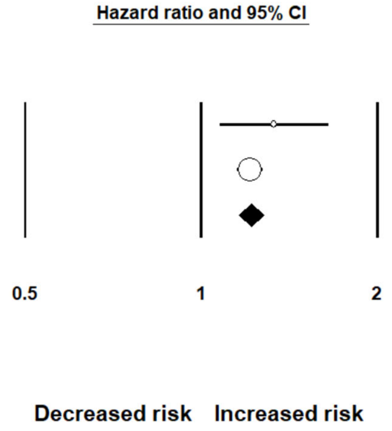

Figure S2. Forest plot and meta-analysis of prospective studies assessing association between higher versus lower consumption of ultra-processed food and depression risk

Table S1. Search terms per database listed below (and the number of studies retrieved).

**MEDLINE** (yielded 433 results)
(("ultra-processed food*" OR "ultraprocessed food*" OR "ultra processed food*" OR "NOVA food classification system" OR UPF)<br><br>AND<br><br>(mental disorder[MeSH Terms] OR "mental disorder*" OR depress* or anxi* OR "common mental disorder*" OR "severe mental illness*"))
**Embase** (yielded 862 results)
((‘ultra-processed food’/exp OR ‘ultraprocessed food*’ OR ‘ultra processed food*’ OR ‘NOVA food classification system’ OR UPF)<br><br>AND<br><br>(‘mental disease’/exp OR ‘mental disorder*’ OR depress* or anxi* OR ‘common mental disorder*’ OR ‘severe mental illness*’))
**Scopus** (yielded 414 results)
(("ultra-processed food*" OR "ultraprocessed food*" OR "ultra processed food*" OR "NOVA food classification system" OR UPF)<br><br>AND<br><br>("mental disorder*"))
Total number of studies yielded = 1709
Total number of studies after deduplication = 1083

Table S2. Critical Appraisal Checklist for Cross-Sectional Studies

Author	Were the criteria for inclusion in the sample clearly defined?	Were the study subjects and the setting described in detail?	Was UPF measured in a valid and reliable way?	Were objective, standard criteria used for measurement of the condition?	Were confounding factors identified?	Were strategies to deal with confounding factors stated?	Was mental health measured in a valid and reliable way?	Was appropriate statistical analysis used?
Amadieu et al	Yes	Yes	Yes	Yes	Yes	Unclear	Yes	No
Ayton et al	Unclear	Yes	No	Yes	No	No	Yes	No
Bonaccio et al	Yes	Yes	No	NA	Yes	Yes	Yes	Yes
Coletro et al	Yes	Yes	Unclear	NA	Yes	Yes	Yes	Yes
Faisal-Cury et al	Yes	Yes	Yes	NA	Yes	Yes	Yes	Yes
Filgueiras et al	Yes	Yes	Yes	Yes	Yes	Unclear	Yes	Yes
Lopes Cortes et al	Yes	Yes	No	NA	Yes	Yes	Yes	Yes
Noll et al	Yes	Yes	Yes	Yes	Yes	Yes	Yes	Yes
Ruggiero et al	Yes	Yes	Unclear	NA	Yes	Yes	No	Yes
Schulte et al	Yes	Yes	No	NA	Yes	Yes	Yes	Yes
Silva et al	Yes	Yes	Unclear	NA	Yes	Yes	Yes	Yes
Werneck et al (2020)	Unclear	Yes	No	NA	Yes	Yes	No	Yes
Werneck et al (2020) (COVID)	Unclear	Yes	No	NA	Yes	Yes	Unclear	Yes
Werneck et al (2021)	Unclear	Yes	No	NA	Yes	Yes	No	Yes
Zheng et al	Yes	Yes	Yes	NA	Yes	Yes	Yes	Yes

Table S3. Critical Appraisal Checklist for Cohort Studies

Author	Were the two groups similar and recruited from the same population?	Were the exposures measured similarly to assign people to both exposed and unexposed groups?	Was UPF measured in a valid and reliable way?	Were confounding factors identified?	Were strategies to deal with confounding factors stated?	Were the groups/participants free of the outcome at the start of the study (or at the moment of exposure)?	Was mental health measured in a valid and reliable way?	Was the follow up time reported and sufficient to be long enough for outcomes to occur?	Was follow up complete, and if not, were the reasons to loss to follow up described and explored?	Were strategies to address incomplete follow up utilized?	Was appropriate statistical analysis used?
Adjibade et al.	Yes	Yes	Yes	Yes	Yes	Yes	Yes	Yes	Yes	NA	Yes
Gómez-Donoso et al.	Yes	Yes	Yes	Yes	Yes	Yes	Yes	Yes	Yes	Yes	Yes

Table S4. Details of exposure and outcome variables and average ultra-processed food consumption by study

Author / year	Exposure variable details	UPF exposure cut-offs	MD exposure cut-offs	UPF outcome variable defined	Average UPF intake
Adjibade et al. 2019 (1)	UPF: Categorical (quartiles) & continuous (10% increase)	Q1 ≤10% vs. Q4 19-76% (weight; % grams/day)	NA	NA	- 32% (kilocalories)<br>- 15% (weight)
Amadieu et al 2021 (2)	UPF: Continuous (% total grams/day, excluding alcoholic beverages)	NA	NA	NA	- 27.8% (total food intake)
Ayton et al 2021 (3)	MD: Categorical (between-group comparison of Anorexia Nervosa [AN], Bulimia Nervosa [BN], Binge Eating Disorder [BED])	NA	NA	Continuous (frequency calculated by asking the patient to describe "a typical food intake per day over the past 2 weeks," including specific mealtimes, binging, and purging")	- AN = 55% (total food intake)<br>- BE = 72% (total food intake)<br>- BED = 69% (total food intake)
Bonaccio et al 2021 (4)	MD: Continuous (each psychometric score was scaled by its standard deviation so that regression coefficients indicated the variation in diet quality for 1 standard deviation change for each measure of psychological distress	NA	NA	Continuous (UPF consumption score ranging from -19 to 19 with higher values indicating an increase)	Not reported
Coletro et al 2021 (5)	UPF: Categorical (dichotomous)	UPF above vs. below the weekly average (not specified)	NA	NA	Not reported
Faisal-Cury et al 2021 (6)	UPF: Continuous (frequency of consumption score ranging from 0 to 35)	NA	NA	NA	Male = 18.9 UPF frequency of consumption score<br><br>Female = 20.4 UPF frequency of consumption score
Filgueiras et al 2019 (7)	UPF: Continuous (select UPF: Sausages and Cookies/biscuit in 100 g, and Soft drinks in 100 ml)	NA	NA	NA	- 3441 (kilocalories/day)
Gómez-Donoso et al. 2019 (8)	UPF: Categorical (quartiles)	- Q1 119 vs. Q4 489 (grams/day – energy adjusted)<br>- <2 vs. ≥4 (servings/day)<br>- <15% vs. >33% (kilocalories)	NA	NA	- 276 (grams/day – energy adjusted)<br>- 3.3 (servings/day)<br>- 24% (kilocalories/day)
Lopes Cortes et al 2021 (9)	MD: Categorical (tertiles)	NA	Low (≤12 points)/moderate (13–18 points) vs. high (≥18 points) perceived stress as per Perceived Stress Scale [PSS-10]	Tertiles (low, moderate and high UPF consumption)	UPF intake in the past week (number of consumed groups x days a week) by low, moderate and high perceived stress groups<br><br>- Low = 6.5<br>- Moderate = 7.1

					- High = 8.6
Noll et al 2022 (10)	UPF: Categorical (tertiles)	1st and 2nd vs. 3rd tertiles of UPF intake (not specified)	NA	NA	493.3 (kilocalories/day)
Ruggiero et al 2020 (11)	MD: Categorical (dichotomous)	NA	- Stress at work sometimes/most times vs. no stress<br>- Stress at work often/always stress at home vs. no stress<br>- Stress at home sometimes vs. no stress<br>- Stress at home most of the time vs. no stress<br>- Stress at home often/always vs. no stress	Continuous (% of total energy intake)	- 17.3 % (kilocalories)<br>- 154.8 (weight)
Schulte et al 2022 (12)	MD: Categorical (dichotomous)	NA	Participants with food addiction vs. no food addiction	Continuous (average scores were created for UPF consumption based on five relevant frequency questions)	Not reported
Silva et al 2021 (13)	UPF: Categorical (tertiles)	Principal component analysis generated lifestyle pattern: High consumption of UPF and low consumption of unprocessed or minimally processed foods. This was categorised according to the distribution of tertiles.	NA	NA	25% (grams/kilocalories)
Werneck et al 2020 (14)	UPF: Categorical (dichotomous)	Joint exposure variable: high vs. low UPF intake (7 d/wk vs. 6 or less d/wk) and high vs. low sitting time (4 hr/d vs. 3.99 or less hr/d)	NA	NA	Not reported
Werneck et al 2020 COVID (15)	MD: Categorical (dichotomous)	NA	Participants with previous diagnosis of depression vs. no depression	Categorical (classified as elevated frequency of UPF intake [or risk behaviour] if participants reported eating at least one UPF five or more days per week)	Not reported
Werneck et al 2021 (16)	UPF: Categorical (dichotomous)	High vs. low UPF intake (7 d/wk vs. 6 or less d/wk)	NA	NA	Not reported
Zheng et al 2020 (17)	UPF: Categorical (quartiles) & continuous (using the median value of each quartile as a continuous variable)	Q1 <37% vs. Q4 ≥73% (total energy)	NA	NA	- 54.9% (total energy)<br>- 1201 (kilocalories/day)

Note UPF: ultra-processed food; MD: mental disorder. For consistency, some values have been altered to one decimal place.

References

1. Adjibade M, Julia C, Allès B, Touvier M, Lemogne C, Srour B, Hercberg S, Galan P, Assmann KE, Kesse-Guyot E (2019) Prospective association between ultra-processed food consumption and incident depressive symptoms in the French NutriNet-Santé cohort. BMC medicine 17 (1):1-13
2. Amadieu C, Leclercq S, Coste V, Thijssen V, Neyrinck AM, Bindels LB, Cani PD, Piessevaux H, Stärkel P, De Timary P (2021) Dietary fiber deficiency as a component of malnutrition associated with psychological alterations in alcohol use disorder. Clinical Nutrition 40 (5):2673-2682
3. Ayton A, Ibrahim A, Dugan J, Galvin E, Wright OW (2021) Ultra-processed foods and binge eating: A retrospective observational study. Nutrition 84:111023
4. Bonaccio M, Costanzo S, Bracone F, Gialluisi A, Di Castelnuovo A, Ruggiero E, Esposito S, Olivieri M, Persichillo M, Cerletti C (2021) Psychological distress resulting from the COVID-19 confinement is associated with unhealthy dietary changes in two Italian population-based cohorts. European journal of nutrition:1-15
5. Coletro HN, de Deus Mendonça R, Meireles AL, Machado-Coelho GLL, de Menezes MC (2022) Ultra-processed and fresh food consumption and symptoms of anxiety and depression during the COVID–19 pandemic: COVID Inconfidentes. Clinical nutrition ESPEN 47:206-214
6. Faisal-Cury A, Leite MA, Escuder MML, Levy RB, Peres MFT (2021) The relationship between ultra-processed food consumption and internalising symptoms among adolescents from São Paulo city, Southeast Brazil. Public Health Nutrition:1-9
7. Filgueiras AR, de Almeida VBP, Nogueira PCK, Domene SMA, da Silva CE, Sesso R, Sawaya AL (2019) Exploring the consumption of ultra-processed foods and its association with food addiction in overweight children. Appetite 135:137-145
8. Gómez-Donoso C, Sánchez-Villegas A, Martínez-González MA, Gea A, Mendonça RdD, Lahortiga-Ramos F, Bes-Rastrollo M (2020) Ultra-processed food consumption and the incidence of depression in a Mediterranean cohort: the SUN Project. European journal of nutrition 59 (3):1093-1103
9. Lopes Cortes M, Andrade Louzado J, Galvão Oliveira M, Moraes Bezerra V, Mistro S, Souto Medeiros D, Arruda Soares D, Oliveira Silva K, Nicolaevna Kochergin C, de Carvalho HdS (2021) Unhealthy Food and Psychological Stress: The Association between Ultra-Processed Food Consumption and Perceived Stress in Working-Class Young Adults. International Journal of Environmental Research and Public Health 18 (8):3863
10. Noll M, Zangirolami-Raimundo J, Baracat EC, da Costa Louzada ML, Júnior JMS, Sorpreso ICE (2022) Life habits of postmenopausal women: Association of menopause symptom intensity and food consumption by degree of food processing. Maturitas 156:1-11
11. Ruggiero E, Esposito S, Costanzo S, Di Castelnuovo A, Cerletti C, Donati MB, de Gaetano G, Iacoviello L, Bonaccio M (2021) Ultra-processed food consumption and its correlates among Italian children, adolescents and adults from the Italian Nutrition & Health Survey (INHES) cohort study. Public Health Nutrition 24 (18):6258-6271
12. Schulte EM, Kral TV, Allison KC (2022) A cross-sectional examination of reported changes to weight, eating, and activity behaviors during the COVID-19 pandemic among United States adults with food addiction. Appetite 168:105740
13. Silva SA, do Carmo AS, Carvalho KMB (2021) Lifestyle patterns associated with common mental disorders in Brazilian adolescents: Results of the Study of Cardiovascular Risks in Adolescents (ERICA). Plos one 16 (12):e0261261

14. Werneck AO, Vancampfort D, Oyeyemi AL, Stubbs B, Silva DR (2020) Joint association of ultra-processed food and sedentary behavior with anxiety-induced sleep disturbance among Brazilian adolescents. Journal of Affective Disorders 266:135-142
15. Werneck AO, Silva DRd, Malta DC, Souza-Júnior PRBd, Azevedo LO, Barros MBdA, Szwarcwald CL (2020) Lifestyle behaviors changes during the COVID-19 pandemic quarantine among 6,881 Brazilian adults with depression and 35,143 without depression. Ciencia & saude coletiva 25:4151-4156
16. Werneck AO, Hoare E, Silva DR (2021) Do TV viewing and frequency of ultra-processed food consumption share mediators in relation to adolescent anxiety-induced sleep disturbance? Public health nutrition 24 (16):5491-5497
17. Zheng L, Sun J, Yu X, Zhang D (2020) Ultra-processed food is positively associated with depressive symptoms among United States adults. Frontiers in nutrition:302
